# Korean Red Ginseng Enhances Neurogenesis in the Subventricular Zone of 1-Methyl-4-Phenyl-1,2,3,6-Tetrahydropyridine-Treated Mice

**DOI:** 10.3389/fnagi.2018.00355

**Published:** 2018-11-06

**Authors:** Sun Ryu, Hyongjun Jeon, Sungtae Koo, Seungtae Kim

**Affiliations:** ^1^Korean Medicine Research Center for Healthy Aging, Pusan National University, Yangsan, South Korea; ^2^Department of Korean Medical Science, School of Korean Medicine, Pusan National University, Yangsan, South Korea

**Keywords:** Parkinson’s disease, Korean red ginseng, neurogenesis, 1-methyl-4-phenyl-1, 2, 3, 6-tetrahydropyridine, subventricular zone, ginseng

## Abstract

Regulation of adult neurogenesis plays an important role in therapeutic strategies for various neurodegenerative diseases. Recent studies have suggested that the enhancement of adult neurogenesis can be helpful in the treatment of Parkinson’s disease (PD). In this study, we investigated whether Korean red ginseng (KRG) can enhance neurogenesis in the subventricular zone (SVZ) of a PD mouse model. To accomplish this, male 8-week-old C57BL/6 mice were injected with vehicle or 20 mg/kg of 1-methyl-4-phenyl-1,2,3,6-tetrahydropyridine (MPTP) four times at 2 h intervals. After the final injection, they were administered water or 100 mg/kg of KRG extract and injected intraperitoneally with 50 mg/kg of 5’-bromo-2’-deoxyuridine-monophosphate (BrdU) once a day for 14 consecutive days. After the last pole test, dopaminergic neuronal survival in the striatum and the substantia nigra (SN), cell proliferation in the SVZ and mRNA expression of neurotrophic factors and dopamine receptors in the striatum were evaluated. KRG administration suppressed dopaminergic neuronal death induced by MPTP in the striatum as well as the SN, augmented the number of BrdU- and BrdU/doublecortin (Dcx)-positive cells in the SVZ and enhanced the expression of proliferation cell nuclear antigen, brain derived neurotrophic factor (BDNF), glial cell derived neurotrophic factor (GDNF), cerebral dopamine neurotrophic factor (CDNF), ciliary neurotrophic factor (CNTF), dopamine receptor D3 (DRD3) and D5 mRNAs. These results suggest that KRG administration augments neurogenesis in the SVZ of the PD mouse model.

## Introduction

Adult neurogenesis is the process through which functional, mature neurons are formed from adult neural precursors in specific brain regions, the subventricular zone (SVZ) and the subgranular zone (SGZ) in mammals (Ming and Song, 2011). The regulation of adult endogenous neurogenesis plays an important role in therapeutic strategies for various neurodegenerative conditions such as Parkinson’s disease (PD), Alzheimer’s disease and stroke (Geraerts et al., 2007; Ryu et al., 2016b) because adult neurogenesis may act as an endogenous repair mechanism in the adult brain (Ming and Song, 2011).

It has been suggested that neurotransmitters and neurotrophic factors play an important role in the regulation of endogenous neurogenesis (Abdipranoto et al., 2008). Recent reports have demonstrated that alteration of neurotransmitter levels in PD patients affects adult neurogenesis in the SGZ as well as SVZ and that increases in adult neurogenesis can repair the dopaminergic system in the nigrostriatal pathway (Borta and Höglinger, 2007). Neurochemical deficit of dopamine suppresses neurogenic activity (Borta and Höglinger, 2007), whereas dopamine-enhancing drugs increase neurogenic activity in the SVZ (Hoglinger et al., 2004; Chiu et al., 2015). Increases in brain derived neurotrophic factor (BDNF) have been found to enhance endogenous neurogenesis in the SVZ and the SGZ (Zhao et al., 2008), while the release of neurotrophic factors such as glial cell-derived neurotrophic factor (GDNF) has been shown to promote neurogenesis and synaptic connectivity (Borta and Höglinger, 2007). In addition to neurotrophic factors, dopamine receptor D3 (DRD3) and dopamine receptor D5 (DRD5) are known to be involved in adult neurogenesis as well as the pathophysiology of PD (Borta and Höglinger, 2007; Chen et al., 2013; Chetrit et al., 2013; Lao et al., 2013; Elgueta et al., 2017).

Korean red ginseng (KRG), which is the steamed root of *Panax ginseng* Meyer, is a valuable herb in Asian countries. Recent studies have shown that KRG exerts positive effects in the brain. Specifically, KRG alleviates the decline of learning and memory in aged mice (Lee and Oh, 2015), suppresses inflammatory cytokines in the brain of a stroke rat model (Lee et al., 2011), increases cognitive function in Alzheimer’s disease patients (Heo et al., 2016) and enhances neuronal survival and development in the nigrostriatal pathway of a PD mouse model (Jun et al., 2015; Kim et al., 2016, 2018). Moreover, it has been reported that ginsenosides, which are components of KRG, enhance cell proliferation. Specifically, ginsenoside Rd was found to promote neural stem cell (NSC) proliferation in the brain of an ischemia animal model (Lin et al., 2012) and to alleviate lead-impaired neurogenesis in the brains of aging rats (Wang et al., 2013). Additionally, ginsenoside Rg1 influenced modulation of the proliferation of progenitor cells in the hippocampus (Shen and Zhang, 2004). However, it is still not clear if KRG administration can enhance neurogenesis in the brain of a PD animal model.

We previously demonstrated that KRG exerts neuroprotective properties and improves motor function in PD models through various mechanisms (Jun et al., 2015; Kim et al., 2016, 2018; Ryu et al., 2016a). In the present study, we investigated whether KRG can modulate neurogenesis in the SVZ and the expression of neurotrophic factors in the striatum using a mouse model of PD induced by 1-methyl-4-phenyl-1,2,3,6-tetrahydropyridine (MPTP).

## Materials and Methods

### Animals and Groups

This study was approved by the Pusan National University Institutional Animal Care and Use Committee and animals were handled in accordance with the current guidelines established by the Pusan National University Institutional Animal Care and Use Committee. Male 8-week-old C57BL/6 mice weighing 20–22 g were purchased from Orientbio Inc. (Seongnam, Korea) and housed at a standard temperature (22 ± 2°C) in a light-controlled environment (lights on from 8:00 AM to 8:00 PM) with free access to food and water.

After a 7-day adjustment period, mice were randomly assigned to four groups (*n* = 11 in each group): a vehicle-injected and water-treated control group (Veh), a vehicle-injected and 100 mg/kg KRG-treated group (KRG), a MPTP-injected and water-treated group (MPTP) and a MPTP-injected and KRG-treated group (MPTP+KRG).

### MPTP and Bromodeoxyuridine Injection and KRG Administration

Mice in the MPTP and the MPTP+KRG groups were injected with MPTP-HCl (20 mg/kg; Sigma, St. Louis, MO, USA) intraperitoneally four times at 2 h intervals (total, 80 mg/kg). Mice in the Veh and KRG groups were injected with vehicle (normal saline) on the same schedule. The KRG extract used in this study was acquired from the Korea Ginseng Corporation (Daejeon, Korea). The KRG extract was diluted with sterilized mineral water. One hour after the first MPTP injection, mice in the KRG and the MPTP+KRG group received oral administration of the KRG extract (100 mg/kg) once a day for 14 consecutive days because this dose was found to have the best neuroprotective effect against MPTP toxicity in our previous study (Jun et al., 2015). Mice in the Veh and the MPTP group were administered the same amount of vehicle (sterilized water) on the same schedule. Immediately after the oral administration, all mice were intraperitoneally injected with 50 mg/kg of 5’-bromo-2’-deoxyuridine-monophosphate (BrdU; Sigma, St. Louis, MO, USA) once a day for 14 consecutive days to detect cell mitosis.

### Pole Test

The pole test was performed by modifying the method established by Abe et al. (2001). Briefly, mice (*n* = 6 in each group) were positioned head downwards near the top of a rough-surfaced wood pole (10 mm in diameter; 55 cm in height) and the time taken to arrive at the floor was recorded. The test was repeated three times at 30 s time intervals and behavioral changes were evaluated according to the average of the three descending times. The tests were conducted 1 day before the first KRG administration (day 0), and 2 h after KRG administration on days 7 and 14.

### Immunohistochemistry

After the last behavioral test, mice (*n* = 6 at each group) were deeply anesthetized by isoflurane (JW Pharmaceutical, Seoul, Korea) and then perfused transcardially with 4% paraformaldehyde (PFA) dissolved in 0.1 M phosphate buffer (PFA). Next, brains were quickly harvested, postfixed in 4% PFA for 24 h, and immersed in 30% sucrose in 0.1 M sodium phosphate buffer (pH 7.4) at 4°C for 3 days. Frozen sections were cut to a thickness of 25 μm using a cryostat (Leica Microsystems, Wetzlar, Germany).

To evaluate dopaminergic neuronal death in the nigrostriatal pathway, sections were placed in normal serum for 2 h at room temperature for blocking. Sections were then incubated with mouse anti-tyrosine hydroxylase (TH, 1:200; Santa Cruz Biotechnology, Santa Cruz, CA, USA) primary antibody overnight at 4°C. After washing, the sections were incubated with Alexa 568-conjugated goat anti-mouse IgG (1:200; Molecular Probes, Eugene, OR, USA) secondary antibody for 2 h at room temperature.

To identify cell proliferation, sections were placed into 2 N HCl at 37°C for 10 min and then 0.1 M boric acid at room temperature for 3 min. After blocking in normal serum for 2 h at room temperature, the sections were incubated with mouse anti-BrdU (1:200; Abcam, Cambridge, UK) and rabbit anti-doublecortin (Dcx; 1:100; Abcam) primary antibodies overnight at 4°C. After washing, the sections were incubated with Alexa 488-conjugated goat anti-rabbit IgG (1:200; Molecular Probes) and Alexa 568-conjugated donkey anti-mouse IgG (1:200; Molecular Probes) secondary antibodies for 2 h at room temperature.

The stained sections were captured with a LSM700 confocal microscope (Carl ZEISS, Oberkochen, Germany). To evaluate the changes in dopaminergic neuronal fibers in the striatum, the mean values of optical density (OD) of the TH in the striatum were determined using Image-Pro Plus 6.0 (Media Cybernetics, Silver Spring, MD, USA). The number of TH-positive neuronal cells in the substantia nigra (SN) was manually counted to evaluate the survival of dopaminergic neurons. Additionally, the numbers of BrdU-positive cells and BrdU/Dcx double-labeled cells in the SVZ were counted manually on each capture. To minimize the possibility of observer bias, an independent observer that did not know the expected results manually counted the cells bilaterally in three continuous striatal sections, and the cell counts were confirmed three times.

### Quantitative Real Time PCR

After the last behavioral test, mice (*n* = 5 at each group) were sacrificed and the bilateral striata were quickly removed, frozen in liquid nitrogen and then homogenized using a Polytron homogenizer. Real time PCR analysis was subsequently conducted to examine the proliferating cell nuclear antigen (PCNA), BDNF, GDNF, cerebral dopamine neurotrophic factor (CDNF), ciliary neurotrophic factor (CNTF), DRD3 and DRD5. Total RNA was isolated using a RNeasy Lipid Tissue Kit (Qiagen, Valencia, CA, USA) and treated with DNase. Reverse transcription was performed using a High-Capacity cDNA Reverse Transcription Kit (Applied Biosystems, Foster City, CA, USA) based on the manufacturer’s protocols. Primer sequences were designed using a software program (Applied Biosystems) and synthesized commercially (Bioneer, Korea). The PCR primers used in this study were as follows: PCNA (forward, 5′-TTTGAGGCACGCCTGATCC-3′; reverse, 5′-GGAGACGTGAGACGAGTCCAT-3′), BDNF (forward, 5′-GGATGAGGACCAGAAGGTTGC-3′; reverse, 5′-TTGTCTATGCCCCTGCAGCCT-3′), GDNF (forward, 5′-ATGGGATGTCGTGGCTGTCTG-3′; reverse, 5′-TCTCTGGAGCCAGGGTCAGAT-3′), CDNF (forward, 5′-GGTCGCTAAAATTGCAGAGC-3′; reverse, 5′-AAGGTAGCCCAGCCCACTAT-3′), CNTF (forward, 5′-GGGACCTCTGTAGCCGCTCTATCTG-3′; reverse, 5′-GTTCCAGAAGCGCCATTAACTCCTC-3′), DRD3 (forward, 5′-TTTGGCAACGGTCTGGTATGT-3′; reverse, 5′-CCAGGCTCACCACTAGGTAG-3′), DRD5 (forward, 5′-TCTGGCCGTCTCAGACCTC-3′; reverse, 5′-GGGTCATCTTGCGCTCGTA-3′) and glyceraldehyde-3-phosphatedehydrogenase (GAPDH; forward, 5′-GGCATTGCTCTCAATGGACAA-3′; reverse, 5′-CCGAGGTTGGGATAGGGCC-3′). GAPDH was used as the reference standard to normalize the expression levels of the target genes. cDNA amplification was performed using the Maxima SYBR Green qPCR Master Mix (Applied Biosystems) and the following conditions: 20 s denaturation at 94°C followed by 40 cycles of 1 min annealing and extension at 60°C in an ABI 7700 sequence detector system (Applied Biosystems).

### Statistical Analysis

The behavioral data of the pole test were expressed as the means ± the standard error of the mean and analyzed by two-way ANOVA (group × time) with Bonferroni’s multiple comparison test. The cell counts, optical densities and mRNA expressions among the four groups were expressed as the means ± the standard deviation and analyzed by one-way ANOVA with Tukey’s multiple comparison tests. Prism five for Windows (GraphPad Software Inc., La Jolla, CA, USA) was used for all statistical analyses and a *p* < 0.05 was considered statistically significant.

## Results

### KRG Administration Alleviates MPTP-Induced Behavioral Dysfunction

**Figure 1:**
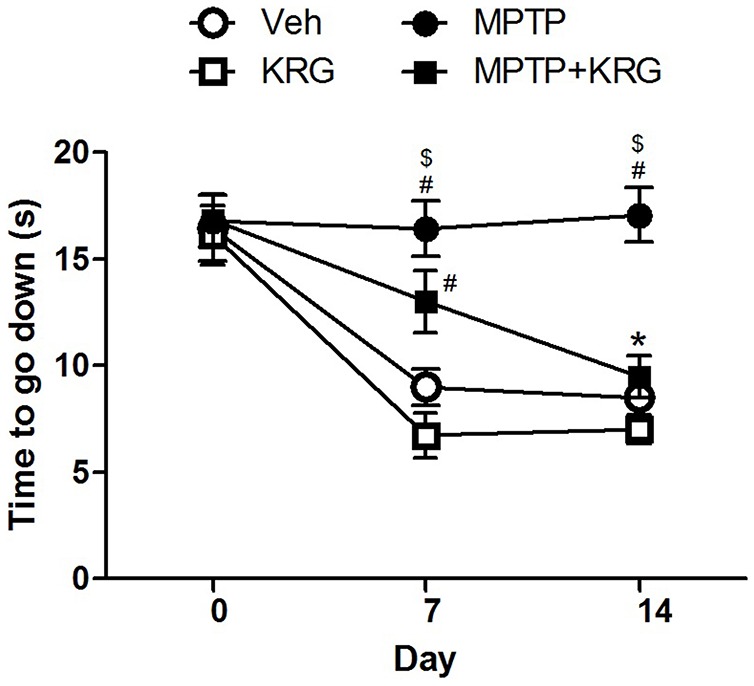
Result of the pole test. On day 7, the descending time in the methyl-4-phenyl-1,2,3,6-tetrahydropyridine (MPTP) group was significantly higher than that in the Veh and the KRG groups and that in the MPTP+KRG group was also significantly higher than that in the KRG group. On day 14, the descending time in the MPTP group was still higher than those of the other groups, but that in the MPTP+KRG group was significantly lower than that in the MPTP group. Data are presented as the means ± the standard error of the mean (*n* = 6 in each group). All results were determined by two-way ANOVA (group × time) with Bonferroni’s multiple comparison test. ^#^*p* < 0.05 compared with the Veh group. ^$^*p* < 0.05 compared with the KRG group. *p < 0.05 compared with the MPTP group. Veh, a vehicle-treated control group; KRG, Korean red ginseng treated group; MPTP, MPTP-injected and vehicle-treated group; MPTP+KRG, MPTP-injected and KRG-treated group.

A pole test was performed to evaluate the influence of KRG administration on motor function. There were no significant differences in the descending times among groups before KRG administration (day 0). On day 7, the descending time in the MPTP group was significantly longer than those in the Veh and the MPTP groups (*p* < 0.05 in each group). Additionally, the descending time in the MPTP+KRG group was lower than that in the MPTP group and higher than those in the Veh and the MPTP groups, but was only significantly different from that in the KRG group (*p* < 0.05). On day 14, the descending time in the MPTP group was significantly higher than those in the other three groups (*p* < 0.05 in each group), but the times were not significantly different among the Veh, MPTP and MPTP+KRG groups (Figure 1). These results indicate that MPTP injection induces motor dysfunction, but KRG administration can alleviate it.

### KRG Administration Suppresses MPTP-induced Dopaminergic Neuronal Loss

**Figure 2:**
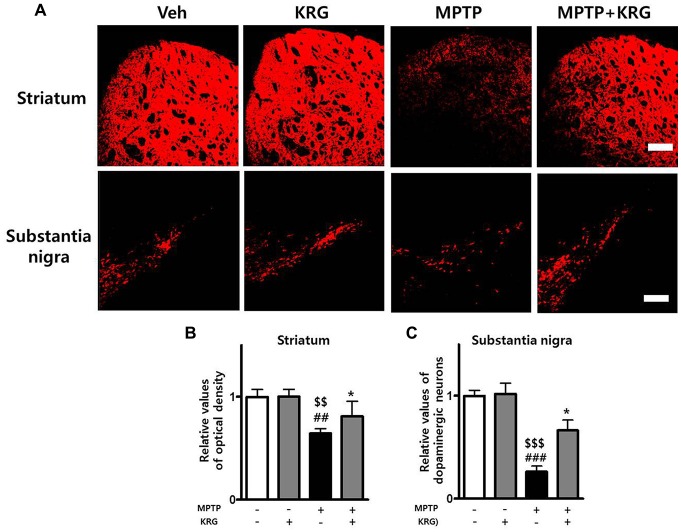
Effects of KRG on MPTP-induced dopaminergic neuronal death in the nigrostriatal pathway. MPTP treatment significantly decreased dopaminergic neurons in the striatum and the substantia nigra (SN), whereas KRG administration significantly suppressed the MPTP-induced dopaminergic neuronal death. **(A)** Tyrosine hydroxylase (TH)-specific immunohistochemical staining in the striatum and the SN. **(B)** Relative value of the optical density (OD) in the striatum. **(C)** Relative value of the number of dopaminergic neurons in the SN. Scale bar: 200 μm. Data are presented as the means ± standard deviation (*n* = 6 in each group). All results were determined by one-way analysis of variance with Bonferroni’s multiple comparison test. ^#^^#^*p* < 0.01 and ^#^^#^^#^*p* < 0.001 compared with the Veh group. ^$$^*p* < 0.01 and ^$$^^$^*p* < 0.001 compared with the KRG group. *p < 0.05 compared with the MPTP group.

The OD of TH-positive neurons in the striatum of the MPTP group was significantly decreased relative to that of the Veh and the KRG groups (*p* < 0.01 in each group). However, the density in the striatum of the MPTP+KRG group was significantly increased when compared to that in the MPTP group (*p* < 0.05). The number of TH-positive neurons in the SN of the MPTP group was significantly decreased relative to those in the Veh and KRG groups (*p* < 0.001 in each group). However, the number in the SN of the MPTP+KRG group was significantly increased when compared to that in the MPTP group (*p* < 0.05, Figure 2). These findings suggest that KRG administration protects dopaminergic neurons in the nigrostriatal pathway from MPTP toxicity.

### MPTP Promotes Neurogenesis in the SVZ

**Figure 3:**
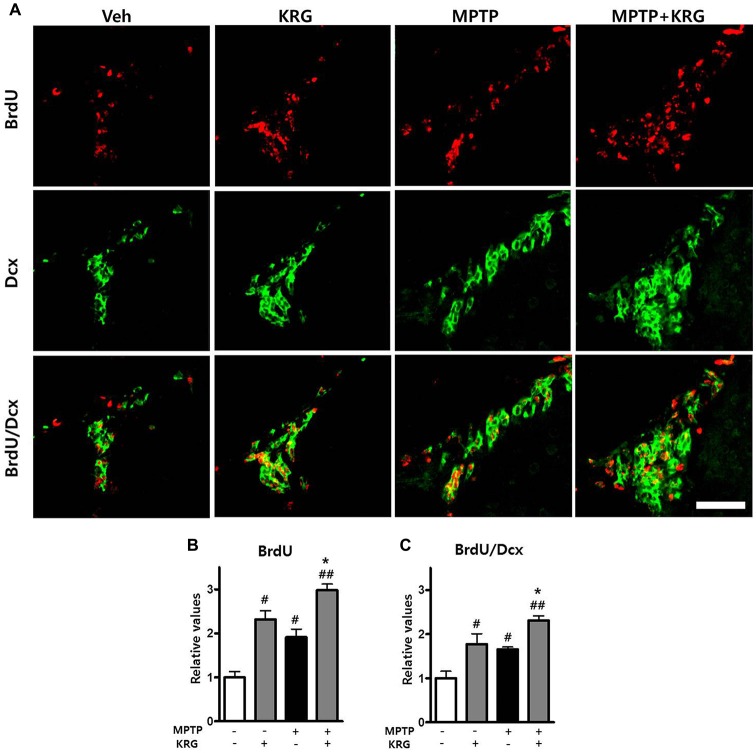
Effects of KRG on cell proliferation in the subventricular zone (SVZ) of the vehicle-or MPTP-treated mice. MPTP promoted proliferation and differentiation of endogenous neural stem cells (NSCs) in the SVZ and KRG administration further enhanced proliferation and differentiation of endogenous NSCs in the SVZ. **(A)** BrdU (red) and Dcx (green)-specific immunohistochemical staining in the SVZ. **(B)** Number of BrdU-positive cells in the SVZ. **(C)** Number of BrdU/Dcx-positive cells in the SVZ. Data are expressed as the mean ± SD (*n* = 6) and were analyzed using one-way analysis of variance with Bonferroni’s multiple comparison test. ^#^*p* < 0.05 and ^#^^#^*p* < 0.01 compared with the Veh group. *p < 0.05 compared with the MPTP group. Scale bars: 50 μm. BrdU, 5-bromo-2′-deoxyuridine; Dcx, doublecortin.

**Figure 4:**
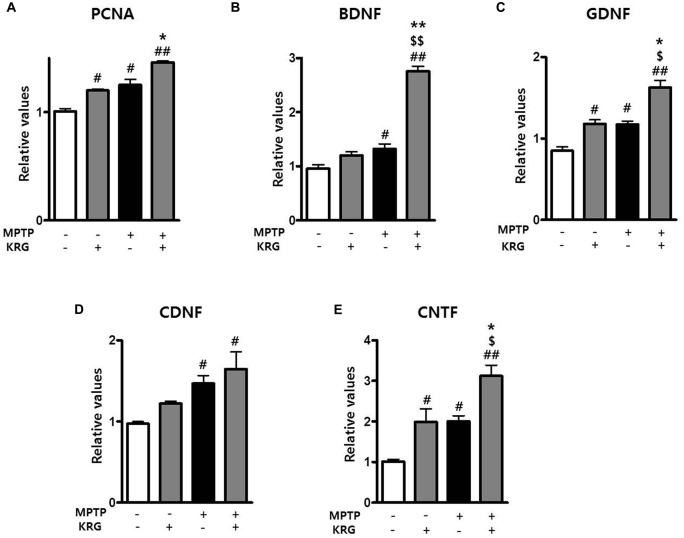
Effects of KRG on neurotropic factors in the striatum of the vehicle-or MPTP-treated mice. Expression of PCNA **(A)**, BDNF **(B)**, GDNF **(C)**, CDNF **(D)** and CNTF **(E)** mRNAs in the SVZ was quantified using real time qPCR. GAPDH was used as an internal control. The data are presented as the mean ± SD of five independent experiments (*n* = 5 in each group). Data were analyzed using one-way analysis of variance with Bonferroni’s multiple comparison test. ^#^*p* < 0.05 and ^#^^#^*p* < 0.01 compared with the Veh group. ^$^*p* < 0.05 and ^$$^*p* < 0.01 compared with the KRG group. *p < 0.05 and **p < 0.01 compared with the MPTP group. PCNA, proliferation cell nuclear antigen;BDNF, brain derived neurotrophic factor; GDNF, glial cell derived neurotrophic factor; CDNF, cerebral dopamine neurotrophic factor; CNTF, ciliary neurotrophic factor.

To detect MPTP-induced cell proliferation in the SVZ, the number of BrdU-positive cells was measured 14 days after MPTP injection (day 14). The number was significantly increased in the MPTP group compared to the Veh group (*p* < 0.05, Figures 3A,B) and the level of PCNA mRNA in the MPTP group was significantly higher than that in the Veh group (*p* < 0.05, Figure 4A).

To analyze the differentiation potential of endogenous NSCs in the SVZ, we performed double labeling with the antibodies against BrdU and against Dcx, a marker for migrating neuronal cells. The number of BrdU/Dcx-positive cells in the MPTP group was significantly higher than that in the Veh group (*p* < 0.05, Figures 3A,C), suggesting that MPTP induces the expansion and differentiation of endogenous NSCs in the SVZ.

### KRG Enhances Neurogenesis in the SVZ

To determine if KRG can enhance cell proliferation in the SVZ, the number of BrdU-positive cells was measured 14 days after MPTP injection. In the KRG group, the number of cells was significantly increased compared to the Veh group (*p* < 0.05), while the number in the MPTP+KRG group was significantly increased compared to the Veh and the MPTP groups (*p* < 0.01 and *p* < 0.05, respectively, Figures 3A,B). To explore the influence of KRG administration on cell proliferation in detail, the level of PCNA mRNA in the SVZ was measured. At 14 days after the KRG treatments, the level of PCNA mRNA in the KRG group was significantly higher than that in the Veh group (*p* < 0.05), while the level in the MPTP+KRG group was significantly higher than those in the Veh and MPTP groups (*p* < 0.01 and *p* < 0.05, respectively, Figure 4A).

To determine if the cells differentiated in response to KRG administration were immature neuronal cells, the number of BrdU/DCX-positive cells was measured in the SVZ. The number of BrdU/DCX-positive cells in the KRG group was significantly greater than that in the Veh group (*p* < 0.05), while the number in the MPTP+KRG group was significantly greater than those in the Veh and the MPTP groups (*p* < 0.01 and *p* < 0.05, respectively, Figure 3C). These findings suggest that KRG administration enhances the expansion and differentiation of NSCs in the SVZ.

### MPTP Increases Neurotropic Factors in the Striatum and KRG Multiples

Growth factors and neurotrophic factors are potent regulators of endogenous adult neurogenesis. Real time qPCR analysis was utilized to investigate changes in neurotrophic factors induced by MPTP and/or KRG in the striatum. Fourteen days after MPTP injection, the mRNA levels of BDNF, GDNF, CDNF and CNTF in the MPTP group were significantly higher than those in the Veh group (*p* < 0.05 for each neurotrophic factor, Figures 4B–E). Additionally, the mRNA levels of GDNF and CNTF in the KRG group were significantly higher than those in the Veh group (*p* < 0.05 in each neurotrophic factor), while the mRNA levels of BDNF, GDNF and CNTF in the MPTP+KRG group were significantly higher than those in the Veh (*p* < 0.01 in each neurotrophic factor), KRG (*p* < 0.01 in BDNF and *p* < 0.05 in GDNF and CNTF) and MPTP (*p* < 0.01 in BDNF and *p* < 0.05 in GDNF and CNTF) groups (Figure 4), suggesting that the KRG would affect the increase of neurogenesis in the SVZ through increases in the neurotrophic factors in the striatum.

### MPTP Increases Dopamine Receptor 3 and 5 in the Striatum and KRG Multiples

**Figure 5:**
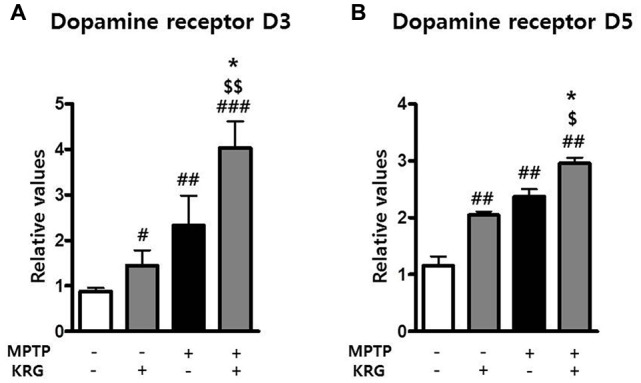
Effects of KRG on dopamine receptors in the striatum of the vehicle-or MPTP-treated mice. Expressions of dopamine receptor D3 (DRD3) **(A)** and D5 **(B)** mRNAs in the striatum were quantified using real time qPCR. GAPDH was used as an internal control. The data are presented as the mean ± SD of five independent experiments (*n* = 5 in each group). Data were analyzed using one-way analysis of variance with Bonferroni’s multiple comparison test. ^#^*p* < 0.05, ^#^^#^*p* < 0.01 and ^#^^#^^#^*p* < 0.001 compared with the Veh group. ^$^*p* < 0.05 and ^$$^*p* < 0.01 compared with the KRG group. *p < 0.05 compared with the MPTP group.

DRD3 and DRD5 are involved in adult neurogenesis and the pathophysiology of PD. To investigate the changes of DRD3 and DRD5 induced by MPTP and KRG, the mRNA expressions of DRD3 and DRD5 in the striatum were evaluated using real-time qPCR. Fourteen days after MPTP injection, the mRNA levels of DRD3 and DRD5 in the MPTP group were significantly higher than in the Veh group (*p* < 0.01 in each dopamine receptor, Figure 4). Additionally, the mRNA levels of DRD3 and DRD5 in the KRG group were significantly higher than those in the Veh group (*p* < 0.01 in each dopamine receptor), while the mRNA levels in the MPTP+KRG group were significantly higher than those in the Veh (*p* < 0.01 in each dopamine receptor) and the MPTP (*p* < 0.05 in each dopamine receptor) groups (Figure 5), suggesting that the KRG would affect the increase of neurogenesis by enhancing DRD3 and DRD5 in the striatum.

## Discussion

This study demonstrated that KRG administration alleviated MPTP-induced behavioral dysfunction and increased cell differentiation and proliferation of NSCs in the SVZ of MPTP-treated mice. Moreover, KRG administration increased the mRNA expressions of the neurotropic factors and the dopamine receptors in the striatum of both vehicle- and MPTP-injected mice.

MPTP-injected mice are a widely used animal model for PD studies. In this study, behavioral functions of mice gradually depreciated and dopaminergic neuronal cell death progressed after MPTP administration. However, KRG administration significantly alleviated MPTP-induced behavioral dysfunction and reduced MPTP-induced dopaminergic neuronal death in the striatum and the SN, indicating that the alleviation of the behavioral impairment with the KRG treatment was due to protection of dopaminergic neurons in the nigrostriatal pathway.

It is not clear whether MPTP enhances or suppress cell proliferation in the SVZ and DG. Hoglinger et al. (2004) reported that MPTP injection resulted in a decrease of PCNA-positive cells among DG and BrdU-positive cells in the SVZ. In contrast, Park and Enikolopov (2010) reported that PCNA-positive cells and BrdU-positive cells increased transiently 14 days after MPTP injection, while Peng et al. (2008) showed that MPTP injection induced dopaminergic neuronal death, but increased striatal neurogenesis. Conversely, chronic MPTP injections (25 mg/kg, bi-weekly for 5 weeks) did not alter cell proliferation in the SVZ (van den Berge et al., 2011). But studies conducted to investigate whether MPTP enhances cell proliferation concluded that the enhancement is a self-repairing process (He and Nakayama, 2009; Park and Enikolopov, 2010). In this study, MPTP injection increased BrdU-positive cells as well as BrdU/Dcx positive cells in the SVZ, as well as PCNA mRNA expression in the striatum; therefore, it is believed that the enhanced neurogenesis in the SVZ in response to MPTP injections may be a compensative mechanism of MPTP-induced cell death.

In the present study, KRG significantly enhanced neurogenesis in the SVZ of vehicle- as well as MPTP-injected mice and significantly increased the levels of BDNF, GDNF, CDNF and CNTF mRNAs in the striatum. The neurotrophic factors are important regulators of adult neurogenesis (Lichtenwalner and Parent, 2006; Zhao et al., 2008); therefore, they can promote neurogenesis and synaptic connectivity (Borta and Höglinger, 2007). Increased BDNF enhanced endogenous neurogenesis in the SVZ and SGZ of the adult brain (Zhao et al., 2008), while GDNF promoted survival of grafted midbrain-derived NSCs in a PD rat model (Lei et al., 2011) and CDNF and CNTF enhanced memory function, neurogenesis and synaptic plasticity in the SGZ (Chohan et al., 2011). These results suggest that KRG would enhance neurogenic activity via modulation of the expression of endogenous neurotrophic factors.

KRG administration increased mRNA expression of DRD3 and DRD5 in the striatum of MPTP-treated mice in this study. Activation of DRD3 reduced the MPTP-induced behavioral impairment and neuronal death (Chen et al., 2013; Elgueta et al., 2017) and enhanced proliferation of neural progenitor cells in the SVZ (Lao et al., 2013). Activation of DRD5 also protected dopaminergic neurons in a PD rat model (Chetrit et al., 2013), promoted neurogenesis in an Alzheimer’s disease mouse model (Shen et al., 2016) and regulated BDNF expression in the rodent brain (Perreault et al., 2013). These results indicate that the KRG-induced increase of DRD3 and DRD5 mRNAs may influence dopaminergic neuronal survival in the nigrostriatal pathway and the increase of neurogenesis in the SVZ.

In conclusion, the present study showed that KRG administration significantly protected against dopaminergic neuronal death in the nigrostriatal pathway and enhanced endogenous adult neurogenesis in the SVZ of MPTP-treated mice by increasing the mRNA expression of BDNF, GDNF, CDNF, CNTF, DRD3 and DRD5. These results suggest KRG would be useful in future strategies for PD treatment via modulation of endogenous adult neurogenesis.

## Author Contributions

SR conducted the immunohistochemistry, quantitative real time PCR and statistical analyses and drafted the manuscript. HJ performed the pole test and assisted with acquisition of immunofluorescent and real time PCR data. SuK assisted with design of the study, coordinated the acquisition of all study data and performed the analysis. SeK designed the experimental setup, interpreted the results, drafted the manuscript and supervised the study. All co-authors were involved in critical revision of the initial drafts.

## Conflict of Interest Statement

The authors declare that the research was conducted in the absence of any commercial or financial relationships that could be construed as a potential conflict of interest.
